# Management of Aphakia with Visual Axis Opacification after Congenital Cataract Surgery Based on UBM Image Features Analysis

**DOI:** 10.1155/2020/9489450

**Published:** 2020-08-06

**Authors:** Wensi Chen, Shiping He, Daoman Xiang

**Affiliations:** ^1^Department of Pediatric Ophthalmology, Guangzhou Women and Children's Medical Center, Guangzhou Medical University, No. 9 Jinsui Road, Tianhe District, Guangzhou 510230, Guangdong Province, China; ^2^Aier Eye Hospital, No. 16 Zhongshan 5th Road, East District, Zhongshan 528400, Guangdong Province, China

## Abstract

**Objective:**

The purpose of this study was to study the imaging features of ultrasound biomicroscopy (UBM) in the aphakia with visual axis opacification (VAO) after congenital cataract surgery.

**Methods:**

From May 2015 to May 2018, aphakia patients with VAO who underwent congenital cataract surgery were examined by high-resolution bag/balloon UBM technique, and the results of UBM imaging were analyzed. According to UBM imaging features, different types of VAO were classified.

**Results:**

A total of 38 children (55 eyes) with VAO were included. 17 patients were bilateral, and 21 patients were unilateral. Patients with VAO were classified into 3 groups according to the UBM imaging features: membranous fibrosis VOA (17 cases, 28 eyes), cortical regeneration VOA (15 cases, 20 eyes), and mixed VOA (6 cases, 7 eyes). The patients in the membranous fibrosis group underwent Nd:YAG laser posterior capsulotomy, those in the cortical regeneration group underwent anterior vitrectomy, and those in the mixed type group underwent anterior vitrectomy with RF capsulotomy tip. After surgery, VAO were removed in all patients. During the follow-up period, in the membranous fibrotic VAO group, iris adhesion and pupillary occlusion were found in 2 eyes, and anterior vitrectomy combined with separation of iris adhesion was performed. In cortical regenerative and mixed type VAO groups, anterior vitrectomy was performed without opacity in the axial region. The total recurrence rate of VAO was 3.46%.

**Conclusion:**

After congenital cataract surgery, the UBM imaging features of aphakia with VAO are helpful to evaluate the condition of VAO before reoperation so as to choose the optimal surgical method to achieve better therapeutic effect.

## 1. Introduction

Visual axis opacification (VAO), also referred to posterior capsule opacification, is a major complication of cataract surgery, especially in infants, which would cause postoperative vision loss [1, 2]. Despite advances in microsurgical techniques, VAO remains a common complication after congenital cataract surgery. In the treatment of congenital cataract in adult cataract surgery, the incidence of VAO is almost 100% [3]. Although the improved incidence of VAO is significantly reduced after the combined posterior capsule incision and anterior vitrectomy, the incidence of VAO in children under 1 year old is still as high as 70.8% [4], which seriously affects the recovery of postoperative visual function in children. Therefore, it is very important for postoperative visual function recovery to accurately assess the transparency of the visual axis during the postoperative period and timely treat according to the evaluation. However, the current postoperative anterior segment examination of children with congenital cataract is usually performed by slit-lamp under nonanesthetic conditions. This type of examination is often limited by the degree of cooperation of the child. Especially for corneal edema and pupillary distraction in children and infants, the effects are very limited.

Ultrasound biomicroscopy (UBM) is a noninvasive UHF ultrasound imaging system that has been widely used in the examination of anterior segmental diseases. Ultrahigh-frequency sound waves can be used to analyze the anterior segment of the living body. At present, UBM research mainly focuses on glaucoma, ocular trauma, and senile cataract. There are few reports in the field of congenital cataract, and there is no report on the value of UBM in VAO clinical evaluation.

The purpose of current study was to study the imaging features of UBM in the aphakia with VAO after congenital cataract surgery and explore the relationship between the types of VAO and surgical methods.

## 2. Methods

### 2.1. Patients

In Guangzhou Women and Children Medical Center, patients underwent congenital cataract extraction surgery from May 2015 to May 2018. The inspection revealed that the visual axis opacity or fundus red light reflection was not clear. These patients underwent parallel UBM examination and finally diagnosed as VAO, and they required surgery again. Patients with secondary glaucoma, corneal opacity, anterior chamber hemorrhage, anterior chamber exudation, iris adhesion, vitreous hemorrhage, and retinal detachment were excluded. A total of 38 patients (55 eyes) were enrolled in this study, including 20 male patients with 28 eyes and 18 female patients with 27 eyes. There were 21 patients with single eye and 17 patients with both eyes. Those patients aged 3–65 months with an average of 12.65 ± 5.77 months. All patients underwent congenital cataract extraction combined with posterior lens incision and anterior vitrectomy. The posterior capsule incision ranged from 3.0 to 4.0 mm in diameter. The anterior vitreous resection ranged from 1/3 of the anterior segment of the visual axis. The preoperative intraocular pressure was in the range of 10–21 mmHg, and no preoperative high intraocular pressure was found. The VAO time after surgery was 19–374 days, with an average of 163.10 ± 89.43 days.

### 2.2. Ultrasound Biomicroscopy

The instrument used for UBM examination is the French Quantel Medical ultrasound biological microscope with MT and 50 MHz water balloon probe. The scanning width was about 16 mm, central axial resolution was about 50 *μ*m, and lateral resolution was 60 *μ*m. All patients were examined in the supine position under oral chloral hydrate sedation. A nurse fixed the patient's head, and the same experienced technician completed the UBM instrument operation to scan the anterior segment of eyes. At least one image perpendicular to the corneal vertex must be obtained in UBM image scanning. All scanned images were saved to the hard drive of the computer system.

### 2.3. VAO Surgery

According to the UBM image characteristics of VAO, the nonsurgical method was selected. The patients in the membranous fibrosis group were treated with Nd:YAG laser lens posterior capsulotomy, the patients in the cortical regeneration group were treated with anterior vitrectomy, and the patients in the mixed group were treated with anterior vitreectomy and electron tear capsule. The children were treated with 0.5% compound tropicamide eye drops (China Shenyang Xingqi Pharmaceutical Co., Ltd.) for 0.5 h before surgery to fully dilute the pupil. After general anesthesia, the position was adjusted followed by surgery.

### 2.4. Nd:YAG Laser Lens Posterior Capsulotomy

The child was placed in a lateral position, and the head was placed in front of a Nd:YAG laser (SuperX, ELLEX Medical Laser, Australia). The operator focused on the aiming light on the opaque area of the posterior capsule of the lens and sequentially cut the turbid posterior capsule along the circular path in a clockwise direction. The laser energy value was 1–3 mJ in single pulse shooting. The posterior capsule incision range is approximately 3.0–4.0 mm in diameter.

### 2.5. Anterior Vitrectomy

The child was placed in a supine position and under surgery under a microscope (Japan Topcon, OMS.800). The bulbar conjunctiva was cut at 3.5 mm from the posterior margin of the cornea. After stopping electrocoagulation, scleral tunnel incision was made 2.5–3.0 mm posterior to the limbus, and viscoelastic (sodium hyaluronate) was injected into the anterior chamber. The 23G vitrectomy head entered the anterior chamber through the scleral tunnel incision, advanced to the posterior capsule of the lens, and excised the opaque posterior capsule of the lens, the cortex of the lens capsule, and the 1/3 vitreous of the anterior segment of the visual axis. The posterior capsule incision range was approximately 3.0-4.0 mm in diameter. The posterior capsule of the lens that cannot be removed by the vitrectomy head needed to be torn by an electronic capsulorhexis and then cut with a vitrectomy head (the electrosurgical needle of the phacoemulsification machine is torn around the center of the posterior capsule) by about 3-4 mm with continuous bubble generation during surgery. Absorb the residual viscoelastic agent in the anterior chamber, suture the scleral incision with a 10–0 absorbable suture, and electrocoagulate the conjunctival incision. After the subconjunctival injection of gentamicin 1 mg and dexamethasone 2 mg, the eye was treated with tobramycin and dexamethasone eye ointment.

### 2.6. Postoperative Follow-Up

Follow-up was performed by the surgeon after surgery to observe the anterior chamber response, the transparency of the visual axis, the red light reflection of the fundus, the complications, and the UBM review. The follow-up time was 6–30 months, and the average follow-up time was 20.53 ± 6.92 months.

### 2.7. Statistical Analysis

All data were statistically analyzed using SPSS 23.0 software (SPSS Inc, Chicago, USA) and expressed as percentage. The data normality was accessed using the Kolmogorov–Smirnov test of normality. Comparison of the difference was assessed by the chi-square test, and *p* < 0.05 was considered statistically significant.

## 3. Results

### 3.1. UBM Image Features

By using the water balloon probe UBM imaging technique, all children with congenital cataract extraction were successfully treated with UBM as early as 7 days after surgery, and a clear image was obtained. According to the analysis of the UBM examination image and the tissue structure involved in the structural variation of the anterior segment of the eyes, the UBM image features of VAO can be summarized into the following three categories.

**Figure 1:**
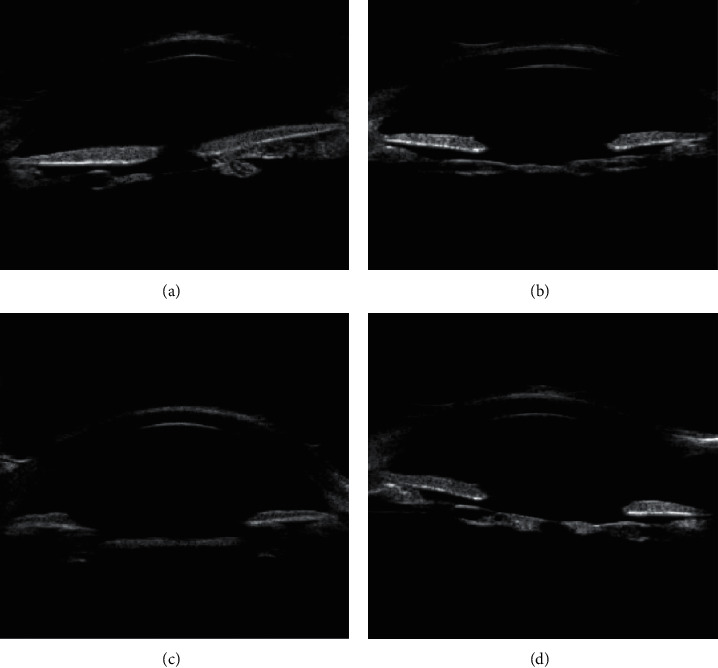
Membranous fibrotic posterior cataract UBM image features: continuous linear or spindle-shaped uneven hyperechoic shadow under the iris (indicated by the arrow).

Type 1 is the membranous fibrotic posterior cataract (Figure 1). UBM image features were summarized as continuous linear or spindle-shaped uneven hyperechoic shadow below the iris pupil area.

**Figure 2:**
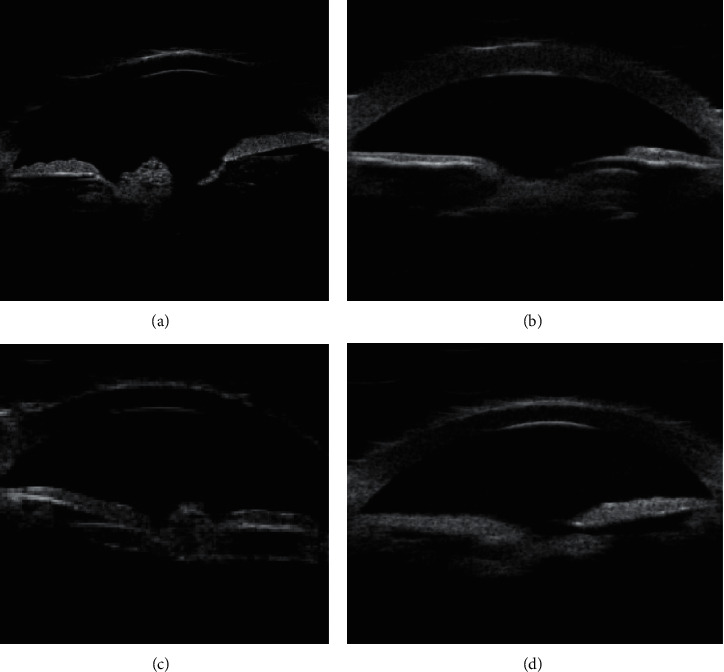
UBM image features of cortical regenerative posterior cataract: irregular echoes or hypoechoic interlacing (indicated by arrows) in the sac or sacral region of the lens capsule.

Type 2 is the cortical regenerative posterior cataract (Figure 2). UBM image features were summarized as intracapsular capsular bag or pupil area, visible irregular, or squamous echoes and low echo interlaced.

**Figure 3:**
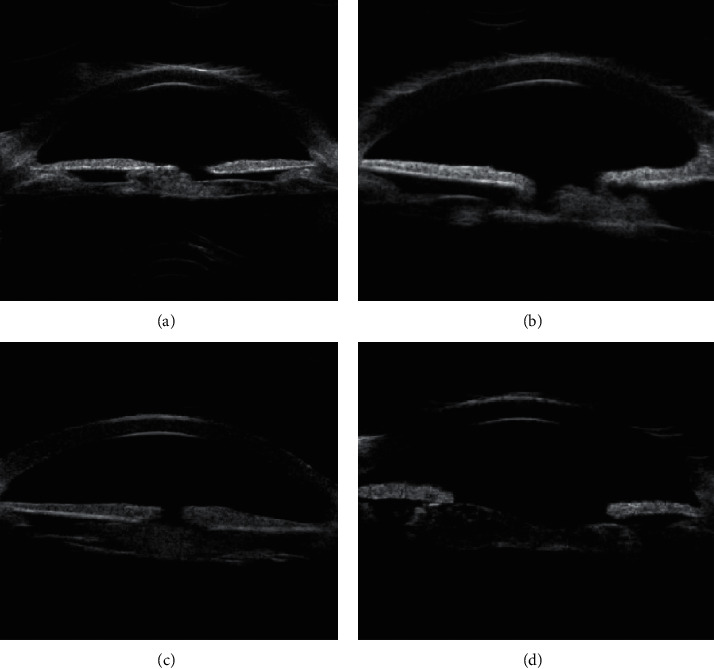
Characteristics of UBM images of mixed posterior cataract: the surface of the anterior and posterior capsules is unevenly thickened, and the echo is enhanced. The irregular lamellae or lumps in the capsular bag are interlaced with the hypoechoic (a and d as indicated by the arrows). According to the arrow, a continuous linear high-echo shadow is visible on the posterior surface of the lens capsule, and the irregular flaky or lumps in the posterior capsule are interlaced with the hypoechoic (b and c as indicated by the arrows).

Type 3 is the mixed posterior cataract (Figure 3). UBM image characteristics were summarized as uneven anterior or posterior capsule lens thickening, echo enhancement, irregular flaky, or lumps in the capsular bag or pupil area. The middle echo was interlaced with the low echo.

Among them, 17 patients (28 eyes) with membranous fibrosis accounted for 50.91%, including 11 patients of both eyes and 6 patients of monocular. 15 patients (20 eyes) with cortical regeneration type accounted for 36.36%, including 5 patients of both eyes and 10 patients of monocular. 6 patients (7 eyes) with mixed type accounted for 12.73%, including 1 patient of both eyes and 5 patients of monocular.

### 3.2. Surgical Methods and Postoperative Conditions

In this study, 28 eyes in the membranous fibrosis group were treated with Nd:YAG laser lens posterior capsulotomy, 20 eyes in the cortical regeneration group and 7 eyes in the mixed group were treated with anterior vitrectomy, and in the mixed group, the electronic capsulorhexis was also performed. The opacity of the optic axis was removed during the operation. The central incision of the posterior capsule of the lens was a 3-4 mm circular transparent zone. The Nd:YAG laser was used to cut the posterior capsule. The posterior capsule cut by vitrectomy showed obvious resection margin, and the success rate of VAO was 100%.

All patients were followed up for 6–30 months. There were no complications such as corneal edema, anterior chamber hemorrhage, elevated intraocular pressure, vitreous hemorrhage, and retinal detachment. In the membranous fibrosis group, UBM was reviewed after surgery, and we found two eyes with iris adhesion and pupillary membrane atresia, and the recurrence rate was 7.14%. The operation was followed by irido-adhesive separation combined with anterior vitrectomy without relapse. After resection in the cortical regenerative group, the UBM visual axis area did not appear turbid again, and the recurrence rate was 0%. The UBM examination results in the mixed group did not show turbidity again in the visual axis area, and the recurrence rate was 0%. The total recurrence rate after VAO was 3.64%.

### 3.3. The Normal Distribution Using the Kolmogorov–Smirnov Test

There are many test methods in statistics, among which the Kolmogorov–Smirnov test can be used to test whether a sample conforms to a known distribution. In SPSS database, a value with *σ x* as standard deviation is a generated normal distribution sample. The difference between *X* and *Y* was tested by the KS test. The results showed the normal distribution *H* = 1 > 0 and *p*=4.45 × 10^−11^ > 0.05. It showed that the distribution in our study was not obeying the normal distribution.

## 4. Discussion

### 4.1. Role of UBM in the Evaluation of Congenital Cataract Surgery

In recent years, attempts have been made to apply UBM examinations to congenital cataract research. Shakankiri et al. [5] studied the role of UBM in the anterior anatomical evaluation of patients with congenital and developmental cataract, and they considered that ultrasound biomicroscopy was a valuable tool for assessing cataract in children and related ocular abnormalities and anterior segment biometrics. Nishijima et al. [6] used UBM examination to evaluate the effect of phacoemulsification and anterior segment morphology after foldable intraocular lens implantation, confirming that UBM contributed to the qualitative and quantitative analysis of the anterior segment structure. Our group studied the imaging characteristics of UBM in congenital cataract in the early stage [7, 8], found that the UBM imaging technique can be used to obtain images of different sections of the anterior segment of the eye, and indirectly obtained a three-dimensional landscape. Personalized abnormalities of the anterior and posterior capsules of congenital cataract, assessment of the opacity of congenital cataract, the condition of the anterior and posterior capsules, and the density of the cortex (nucleus) are conducive to the abnormality of the lens before congenital cataract extraction. A more complete assessment and guidance for the surgeon to choose the appropriate surgical approach is important to reduce intraoperative complications and improve surgical outcomes.

On this basis, we applied the water balloon probe UBM imaging technology to study the VAO of crystal formation after congenital cataract extraction. We found that VAO showed many specific changes after congenital cataract extraction and could be classified according to its specific UBM image characteristics. This study proposes to improve the VAO classification based on the characteristics of UBM images of VAO after congenital cataract extraction. The VAO after congenital cataract surgery was divided into three types (membranous fibrosis, cortical regeneration, and mixed). To the best of our knowledge, this is the first international classification of VAO based on imaging features after congenital cataract extraction. Clinicians can decide to use Nd:YAG laser posterior capsule incision according to different types of VAO characteristic UBM images.

**Figure 4:**
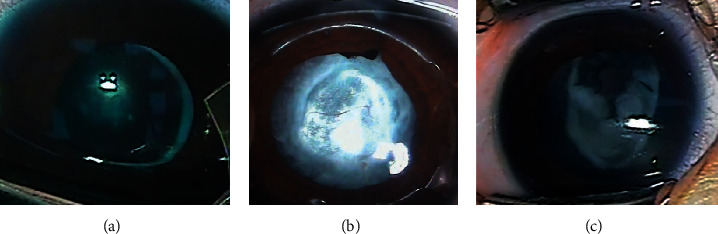
Microscopy of three types of posterior cataract surgery: membranous fibrosis (a), cortical regeneration (b), and mixed (c).

In the membranous fibrosis type of posterior cataract, only the opaque posterior capsule of the pupil area can be observed under the operating microscope (Figure 4(a)). The evaluation of the condition under the iris is limited, and the UBM examination can not only find the central part of the pupil axis outside the thickness and extent of turbidity but also understand the condition of the capsule below the iris and its anatomical relationship with the surrounding tissue. For this type, UBM images showed different degrees of morphological changes in the lens capsule under the iris. We speculated that this morphological change may be related to the proliferation of the lens equatorial epithelial cells, the ability to fibrosis because of infants. Children's tissue is delicate, and inflammatory reaction after cataract surgery is heavy, fibrin exudation in the eye and easy formation of fibrous membrane, providing a scaffold for lens epithelial cell proliferation and migration, which together lead to linear or spindle-shaped fibrillar changes on the posterior capsule. A membranous fibrotic posterior cataract can be diagnosed when a characteristic image of a continuous linear shape or a spindle-shaped uneven hyperechoic image below the iris pupil region appears on the UBM image. It is suggested that this type of VAO can be considered to use a Nd:YAG laser to cut the opaque lens posterior capsule.

The UBM image of the cortical regenerative posterior cataract showed that the irregular lamellae or lumps of the lens residual sac were interlaced with the hypoechoic, which was similar to the opacity of the lens, and some of them have protruded from the residual lens of the lens in the boresight area. We speculated that this type was related to the regeneration of lens cortex. The residual epithelial cells migrate from the periphery to the center and form a cluster of proliferating cells on the surface of the posterior capsule. As time goes on, it gradually accumulates and thickens to form a regenerated cortex, causing posterior cataract. Relative to the image acquired by the surgical microscope (Figure 4(b)), this type of UBM image provides more information. In addition to the white opacity in the pupil area seen under the microscope, it is also possible to determine whether there is a hyperplastic cortex in the visual axis opacity and evaluate its thickness, the degree of density, the scope, and its relationship with surrounding organizations. Because this type of case regenerates the lens cortex and blocks the pupil axis of the pupil, it is necessary to treat VAO through anterior vitrectomy, resected cortical resection, and lens residue.

The mixed posterior cataract surgery microscope image (Figure 4(c)) showed only dense white opacity, and the surgeon could not rely on this image to make an accurate judgment of the turbid tissue. However, UBM images showed abnormal morphological changes in the lens capsule and in the capsular or pupil area, suggesting that the formation of this type of VAO is more complicated, including residual capsule thickening and cortical hyperplasia. We speculated that it may be related to the mitotic proliferation of the lens equatorial epithelial cells and the proliferation of newly produced cortex. It is also related to the small or irregular lens capsule tear, the lens capsule fibrosis, and postoperative inflammatory reaction. This type of VAO can be used to remove the regenerated cortex and cut the proliferating lens capsule through the anterior vitreous cleavage combined with the electronic capsulorhexis technique. It also removes the inflammatory exudate cells and eliminates the epithelial migration of the lens. The stimulating effect and the scaffold function reduce the risk of turbidity in the visual axis. According to this classification, different surgical methods are decided according to different types of VAO characteristic UBM images.

### 4.2. Selection of Different Types of Posterior Cataract

At present, there are two main surgical methods for the treatment of VAO [9, 10]. One is Nd:YAG laser lens posterior capsulotomy, and the other is anterior vitrectomy. Nd:YAG laser lens posterior capsulotomy is the most important method for the treatment of posterior cataract. It has the advantages of short operation time and small damage. However, it is not fully applicable to all patients with posterior cataract, especially in infants with posterior cataract [11]. Infants in neonatal intensive care unit (NICU) have greater chances of developing sensorineural hearing loss (SNHL) because of the presence of multiple risk factors [12]. It has been reported that hereditary hyperferritinemia cataract syndrome (HHCS) should be considered in the differential diagnosis of childhood hyperferritinemia, especially in the presence of normal transferrin saturation [13]. In some infants and young children due to the dense opacity of the posterior lens capsule, it is often necessary to increase the laser energy or increase the number of firings, which may aggravate the degree of damage of corneal endothelial cells and iris tissue and increase the incidence of complications [14]. Clinically, for patients who are only examined using a slit-lamp microscope or a surgical microscope, it is usually only possible to see the opacity of the two-dimensional planar pupil region, and it is not possible to distinguish whether the opacity is membranous or cortical residue or hyperplasia. Under this circumstance, the uniform use of Nd:YAG laser for the treatment of VAO may result in severe anterior segment inflammatory reaction after treatment, which will accelerate the proliferation of lens epithelial cells and migrate in the posterior capsule incision to form a mechanical membrane. Studies have reported that 30%–41% of children with posterior cataract undergoing Nd:YAG laser lens posterior capsulotomy after cataract extraction require surgery again [15]. Anterior vitrectomy, now routinely using a phacoemulsification vitrectomy system, removes the opaque posterior capsule and part of the anterior segment of the vitreous and simultaneously removes lens remnants, inflammatory exudate cells, and some fibrotic lens epithelial cell proliferation. Some studies have also pointed out that the anterior vitrectomy may increase the incidence of retinal detachment and cystoid macular edema [16].

Because of different advantages and disadvantages of different treatments of VAO, preoperative evaluation of VAO after congenital cataract extraction is very important. This study classified VOB-specific UBM image features and selected different surgical treatment methods according to different classifications. For the membranous fibrosis group, because the opaque crystal posterior capsule is thin, a simple and rapid Nd:YAG laser lens posterior capsulotomy can be used to achieve a good surgical success rate. In this study, membranous fibrotic VAO was treated with Nd:YAG laser, and only 2 eyes recurred after surgery. The recurrence rate was 7.14%, which was much lower than the 30%–41% reported in the previous literature. Moreover, it can avoid mechanical damage to the anterior chamber again, shorten the operation time, reduce the risk of anesthesia, reduce the operation cost, and reduce the postoperative inflammatory reaction. However, during surgery, attention should be paid to fully smash the incision posterior capsule fragments to prevent them from adhering to the pupil area, causing irritation of the iris inflammatory reaction, forming a mechanized membrane in the pupil area, and again affecting the transparency of the visual axis.

In summary, by analyzing the UBM-specific image features of VAO, a clearer understanding of the morphological features of VAO can help to improve the predictability of surgery, so that surgeons can choose the best surgical method according to the different types of VAO to achieve better therapeutic results.

### 4.3. Study Limitations

First, because the patients included in the study were all less than 3 years old, their visual acuity could not be assessed objectively. Second, because of the low incidence of congenital cataracts and the time span required for case accumulation, the sample size in this study was small, especially in mixed type. We will further accumulate relevant cases in future research.

In conclusion, by analyzing UBM-specific image features, we first proposed that based on UBM imaging technology, accurate disease assessment and imaging classification of VAO after congenital cataract extraction can be performed. This method is different from the current international classification method and can provide multidimensional high-resolution characteristic structural information, so as to more clearly understand the morphological characteristics of VAO and determine the nature of VAO through morphological features.

## Data Availability

The data used in the study are included in the article.
